# Default Mode Network Connectivity in Stroke Patients

**DOI:** 10.1371/journal.pone.0066556

**Published:** 2013-06-18

**Authors:** Anil Man Tuladhar, Liselore Snaphaan, Elena Shumskaya, Mark Rijpkema, Guillén Fernandez, David G. Norris, Frank-Erik de Leeuw

**Affiliations:** 1 Department of Neurology, Radboud University Nijmegen Medical Centre, Donders Institute for Brain, Cognition, and Behavior, Centre for Neuroscience, Nijmegen, The Netherlands; 2 Radboud University Nijmegen, Donders Institute for Brain, Cognition and Behaviour, Donders Centre for Cognitive Neuroimaging, Nijmegen, The Netherlands; 3 Radboud University Nijmegen, Donders Institute for Brain, Cognition and Behaviour, Centre for Cognition, Nijmegen, The Netherlands; 4 Department of Nuclear Medicine, Radboud University Nijmegen Medical Centre, Nijmegen, The Netherlands; 5 Erwin L. Hahn Institute for Magnetic Resonance Imaging, University of Duisburg-Essen, Essen, Germany; 6 MIRA Institute for Biomedical Technology and Technical Medicine, University of Twente, Enschede, The Netherlands; University of California, San Francisco, United States of America

## Abstract

The pathophysiology of episodic memory dysfunction after infarction is not completely understood. It has been suggested that infarctions located anywhere in the brain can induce widespread effects causing disruption of functional networks of the cortical regions. The default mode network, which includes the medial temporal lobe, is a functional network that is associated with episodic memory processing. We investigated whether the default mode network activity is reduced in stroke patients compared to healthy control subjects in the resting state condition. We assessed the whole brain network properties during resting state functional MRI in 21 control subjects and 20 ‘first-ever’ stroke patients. Patients were scanned 9–12 weeks after stroke onset. Stroke lesions were located in various parts of the brain. Independent component analyses were conducted to identify the default mode network and to compare the group differences of the default mode network. Furthermore, region-of-interest based analysis was performed to explore the functional connectivity between the regions of the default mode network. Stroke patients performed significantly worse than control subjects on the delayed recall score on California verbal learning test. We found decreased functional connectivity in the left medial temporal lobe, posterior cingulate and medial prefrontal cortical areas within the default mode network and reduced functional connectivity between these regions in stroke patients compared with controls. There were no significant volumetric differences between the groups. These results demonstrate that connectivity within the default mode network is reduced in ‘first-ever’ stroke patients compared to control subjects. This phenomenon might explain the occurrence of post-stroke cognitive dysfunction in stroke patients.

## Introduction

The majority of stroke survivors suffer from post-stroke cognitive impairment. These cognitive deficits after infarction are usually well explained by size and location of the infarction [1]. However, occasionally the post-stroke cognitive symptoms cannot be explained by the location of the infarction, rather they are attributable due to impairment of cortical regions remote from the lesion. A possible explanation for these remote effects can be the disruption of neuronal input vital to the function of that remote cerebral region or of a certain network [2], [3]. An illustration of such a remote stroke effect is our recent finding of reduced medial temporal lobe (MTL) functionality in stroke patients with an impaired episodic memory performance, but without an infarction in the MTL [4]. To date, the underlying pathophysiology for this reduced functionality is not known.

The MTL is a part of the default mode network (DMN) that also comprises of posterior cingulate cortex, medial prefrontal cortex and bilateral parietal cortices [5]–[7]. The cortical regions of the DMN frequently deactivate during active tasks [8], while during wakeful resting condition they are active [9]. This network is important in episodic memory processing [6], [10] and is altered in neurodegenerative diseases, like Alzheimer’s disease [6] and mild cognitive impairment [11]. This supports the notion that this underlying network is disrupted in disorders affecting episodic memory processing. This network is also implicated in self-referential and reflective activity, and to internal and external stimuli. We therefore investigated, as an extension of our previous study [4], the functional connectivity of the DMN in stroke patients during resting state condition, in order to investigate the brain network properties.

A novel approach in understanding the intrinsic state of brain activity is the study of the spatiotemporal patterns of the blood-oxygen level-dependent (BOLD) fluctuations in the task-independent condition [12]. Several spatially distinct cortical regions exhibit similar temporal properties of the spontaneous ongoing BOLD fluctuations that determine the so-called resting state network (RSN) [13]. These temporally synchronized cortical regions are considered to be functionally connected, mediated by the direct and indirect anatomical connections [14], [15]. The DMN is a robust RSN that has been consistently demonstrated in both healthy control subjects and patients.

In this study, we performed independent component analysis (ICA) to identify the resting-state DMN and region-of-interest (ROI) analysis to examine the inter-regional functional connectivity between the regions of DMN as the independent component may not capture all the variance of the data. We hypothesized that patients following an infarction would have an altered DMN compared to the control subjects. More specifically, the functional connectivity between the key regions involved in DMN (including MTL) would be reduced in the patient group compared with controls.

## Materials and Methods

### Study Population

Acute ischemic stroke patients, admitted at the Department of Neurology of the Radboud University Nijmegen Medical Centre between September 2005 and May 2007, were considered for enrollment in this study. The selection of patients and controls has been described in detail before [4]. In short, patients were eligible when diagnosed with acute stroke, defined as sudden occurrence of acute neurological symptoms that can only be explained by occlusion of a specific artery with a compatible ischemic lesion visible on CT or MRI-scan. Exclusion criteria were age above 75 years, pre-existent cognitive decline as assessed by the short Informant Questionnaire on cognitive decline in elderly (IQCODE) [16] and concurrent cognitive dysfunction defined as a score less than twenty-four on mini mental state examination (MMSE) [17], history of concomitant neurological disease, presence of disease or use of medication that could affect cognition or MRI disruptions, history of drug or alcohol abuse, presence of severe white matter lesions (defined as a score ≥2 in two or more regions as assessed by Age-Related White Matter Changes scale [18]), clinical or radiological evidence of a previous infarction, aphasia and MRI contraindications. These exclusion criteria were chosen to reduce the probability of reduced pre-stroke DMN activity in stroke patients. Healthy control subjects closely matched to the patient group for age, gender and education participated in this study [4]. Control subjects were excluded if they had pre-existent cerebrovascular disease such as silent brain infarctions and/or pre-existent cognitive problems, that could affect episodic memory function and DMN activity [4].

### Neuropsychological Assessment

All subjects underwent an extensive neuropsychological assessment between six and eight weeks after stroke onset that included an MMSE, IQCODE, Hospital Anxiety Depression Scale [19], California verbal learning test (CVLT) [20] and standard neurological examinations. Furthermore, clinical severity of all patients was rated according to the National Institute of Health Stroke Scale (NIHSS) at admission. Functional and physical status was determined by the modified Barthel index [21] and modified Rankin Scale [22].

### Magnetic Resonance Imaging

Magnetic resonance imaging was performed on a 1.5 Tesla Siemens Magneton Sonata scanner (Siemens Medical Solutions, Erlangen, Germany) which included T1 3D magnetization-prepared rapid gradient-echo (MPRAGE) imaging (time repetition [TR] = 2.25 sec, time echo [TE] = 3.68 msec, flip angle [FA] = 15°, 176 1 mm slices, 256×256 matrix, field of view [FoV] = 256 mm) and resting state data using a gradient-echo echo planar imaging (EPI) sequence (TR = 2.4 sec, TE = 30 msec, FA = 90°, 35 slices, 64×64 matrix, voxel size = 3.5×3.5×4.0 mm^3^, 300 volumes, FoV = 224 mm). All participants were scanned on the same scanner.

All patients underwent MRI scanning nine to twelve weeks after stroke onset. Twelve minutes of resting state scanning was performed before the n-back working memory paradigm. The results of the N-back paradigm have been published [4] and will not be reported in this study. Subjects were instructed to close their eyes, think of nothing in particular and relax while avoiding falling asleep. None of the subjects reported falling asleep during scanning.

### Data Analysis

Image processing and statistical analysis was performed using the statistical parametric mapping software SPM5 (http://www.fil.ion.ucl.ac.uk/spm/). The first five resting state images were discarded because of the possible spin saturation effects. Remaining images were motion corrected and the mean image was coregistered to each subject’s T1-weighted image. Subsequently, the images were normalized to the Montreal Neurological Institute (MNI) T1 template and resampled at an isotropic voxel size of 2 mm. To reduce the effects of low and high non-neuronal noise, the images were then temporally band-pass filtered between 0.01 Hz and 0.1 Hz using fifth order Butterworth filter. Finally, the images were spatially smoothed using a Gaussian kernel of 8 mm full width at half maximum (FWHM).

### Independent Component Analysis

The independent component analysis (ICA) was performed using Group ICA of fMRI Toolbox (GIFT, http://icatb.sourceforge.net/version 2.0c) [23] to identify the task-independent network activity in the group of the subjects. ICA is a model-free method that can identify distinct components capturing spatially independent and temporal synchronous patterns of the resting state data and, as such, can distinguish different RSN’s. The analyses were conducted on the data of both control subjects and patients together to identify RSN’s common to both groups. Twenty independent components were estimated as the optimal number of components. Group maps were obtained using one-sample *t-*tests using a threshold of P<0.05 family wise error (FWE)-corrected for multiple comparisons. For the group comparisons, two sample t-tests were applied with a height and extent threshold of P<0.05 [24], corrected at the whole-brain level, masked by the group maps (thresholded at P<0.05, uncorrected) to ensure that only voxels involved in the specific resting state network are evaluated. The default mode component was selected using the GIFT toolbox in which all components were spatially correlated with DMN spatial template. This template was generated by WFU Pick-atlas [25], which included Brodmann’s area 7, 10, 39, 23 and 31. The component with the highest spatial correlation was considered as the default mode component [6]. In this study, we investigated DMN because of our a priori hypothesis that stroke patients would have reduced DMN activity. Also, we calculated the power spectral density of the time course of the DMN with fast Fourier transform for every subject to test whether the differential spatial pattern of the DMN was due to the alterations in the frequency distribution of the time course. The component containing the cerebellum was considered a control component for which we did not expect to find any group differences, as there were no infarctions in the cerebellum.

### ROI-based Analysis

ROI-based analyses were performed to investigate the inter-regional functional connectivity of the default mode network to examine the direct functional connectivity between the pre-specified region and other regions. Various non-neural fluctuations were removed from the data, through multiple regression technique [7], which included the six motion parameters, the quadratic and cubic effects of the motion parameters [26], the mean time course of the white matter and cerebrospinal fluid along with their temporal derivatives. The ROI’s were determined by the results of the group-analysis of independent component depicting the DMN (Table 1). A sphere of 6 mm was created centered on the peak voxels of every region. Subsequently, the first eigenvariate of the time course within these ROI’s was extracted and Pearson’s correlation coefficients was calculated for every pair-wise region, which were then converted to *z*-values by applying a Fisher’s *r*-to-*z* transformation. The differences in correlation between the groups were determined by applying two-sample *t*-test with a threshold of P<0.05 on the pair-wise region that was significantly different from zero using one-sample *t*-test at the threshold of P<0.05 [27]. To control for the multiple comparisons, FDR-correction (with a q-value of 0.05) was applied.

**Table 1:** Peak voxels for the default mode network component.

Location	CS	Anatomy	Hemisphere	MNI			T
				x	y	z	
Parietal	9438	Precuneus/posterior cingulate gyrus	R/L	−4	−60	16	30.03
	996	Inferior parietal lobe	R	52	−58	18	14.64
	887	Inferior parietal lobe	L	−50	−70	24	9.61
Frontal	1483	Middle prefrontal gyrus	R/L	4	60	4	12.61
	287	Superior frontal gyrus	R	22	32	48	10.64
	373	Superior frontal gyrus	L	−26	26	46	9.43
Temporal	575	Medial temporal lobe	R	26	−28	−14	7.13
	302	Medial temporal lobe	L	−28	−30	−14	7.79
	286	Middle temporal gyrus	R	64	−4	−18	10.51
	119	Middle temporal gyrus*	L	−64	−12	−16	5.49

Peak voxels of the regions for the independent component depicting the default mode network. These peak voxels were used for the region of interest (ROI) based analysis.

* Left middle temporal gyrus was only significant at a lower threshold; this was obtained at a threshold of P<0.001 false-discovery rate (FDR) corrected. CS = Cluster size, R = Right, L = Left, MNI = Montreal Neurological Institute.

### Brain Morphology

Automated segmentation was conducted on the high-resolution T1-weighted anatomical image using SPM5. Independent two-tailed *t-*tests were performed on the gray matter, white matter, cerebrospinal fluid and total intracranial volumes to determine the statistical significance for the group comparisons. In addition, optimized voxel-based morphometry [28] was carried out to investigate the regional volumetry differences of the gray matter between stroke and control subjects. The segmented, normalized and modulated, gray matter images were smoothed using a Gaussian kernel of 8 mm FHWM. Analysis of covariate (ANCOVA) was then applied including total intracranial volume, age, gender and education as nuisance variables. The group differences were statistically assessed by two-sample *t*-test using a threshold of P<0.05 FDR-corrected**.** Furthermore, manual segmentation was conducted using the interactive software program ‘ITK-SNAP’ (http://www.itksnap.org) to estimate the hippocampal volumes more precisely. This was done in a standardized way according to the previously published protocol [29]. Intraclass interrater correlation coefficients were 97% and 96% for the left and right hippocampus respectively.

### Statistical Analysis for Clinical Data

Independent *t*-tests were applied on the normal distributed baseline characteristics and neuropsychological scores, Chi-square tests for nominal data and Mann-Whitney U-analyses for the non-Gaussian distributed data.

### Ethics Statement

The study protocol was approved by the medical ethics committee region Arnhem-Nijmegen and all the participants gave written informed consent according to the Declaration of Helsinki. None of the participants had a compromised capacity or ability to consent, which was established by neurological examination.

## Results

### Study Population Characteristics

Data were available of 49 subjects, of whom eight subjects were excluded because of the use of different resting state protocol (n = 7) and absence of resting state data (n = 1). Twenty-one healthy control subjects (52% male; mean age 51 years SD 12) and twenty stroke patients (65% male; mean age 55 years SD 14) were included. Table 2 presents the demographic and neuropsychological characteristics of the study population. Stroke patients performed significantly worse than the control subjects only on the delayed recall task of the CVLT and on the MMSE. Infarctions were located in the internal capsule (four left and one right), in the corona radiata (one left and three right), in the thalamus (one left and one right), in the occipital lobe (two left and two right), in the brainstem (four) and in the parietal lobe (one right).

**Table 2:** Demographic and neuropsychological characteristics of stroke and control subjects.

	Stroke (n = 20)	Control (n = 21)
Age, years (SD)^a^	55.1 (11.8)	51.3 (13.7)
Education (range)^b^	5 (4–7)	5 (2–7)
Gender, males (%)^c^	13 (65.0)	11 (52.4)
Right handedness (%)^c^	81	89
Left-sided infarction (%)	53	NA
MMSE^b^	28 (25–30)	29 (28–30)
Anxiety^b^	5 (0–14)	4 (0–12)
Depressive symptoms^b^	4 (0–14)	4 (0–13)
CVLT – delayed recall (SD)^a^	8.7 (4.9)	12.3 (2.0)
Modified Barthel index^a^	20 (0.0)	20 (0.0)
Modified Rankin scale	1 (0–3)	NA
NIHSS score	2 (1–16)	NA

Values represent means (SD), median (range), or proportion (%). Stroke patients performed significantly worse on MMSE (p = 0.038) and CVLT-delayed recall score (p = 0.034). On other neuropsychological tests, no significant differences were found. Education score of 5 means 10–11 years of education. MMSE = Mini Mental State Examination, CVLT = California Verbal Learning Test, NIHSS = National Institutes of Health Stroke Scale. NA = not applicable.

a Independent-samples t test.

b Mann-Whitney test.

c Chi-square analysis.

### ICA

**Figure 1:**
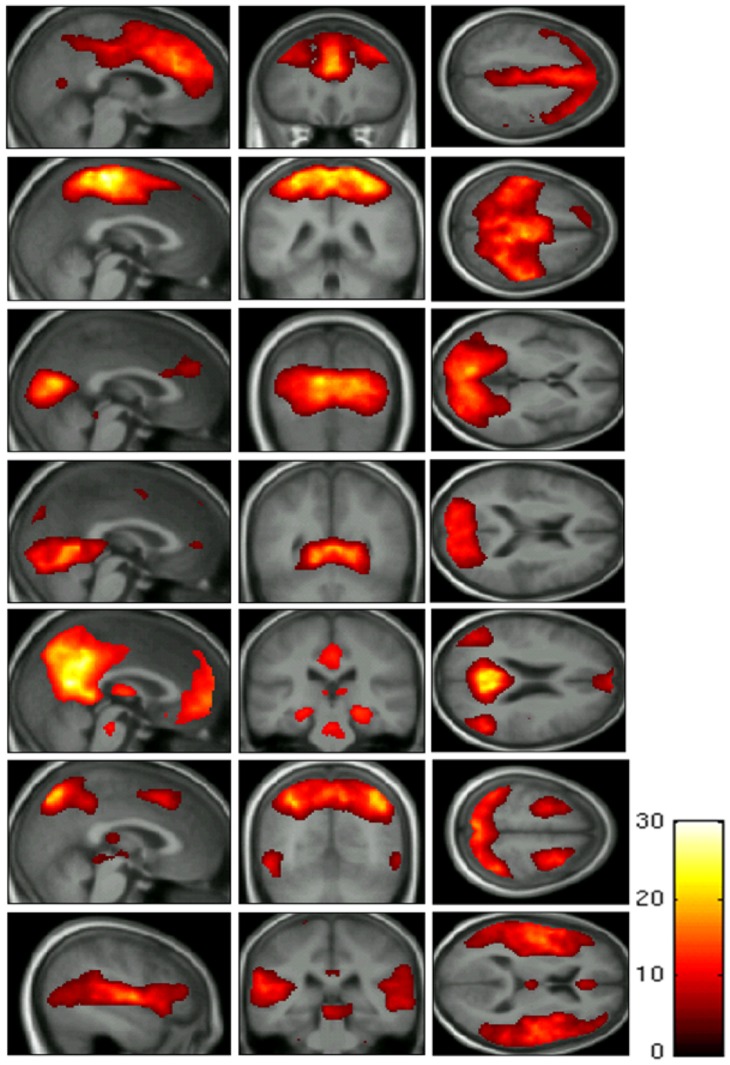
Group analyses of the independent component analysis revealing seven distinct resting state networks (default mode, dorsal attention, auditory, frontal, sensorimotor lateral and medial visual network).

**Figure 2:**
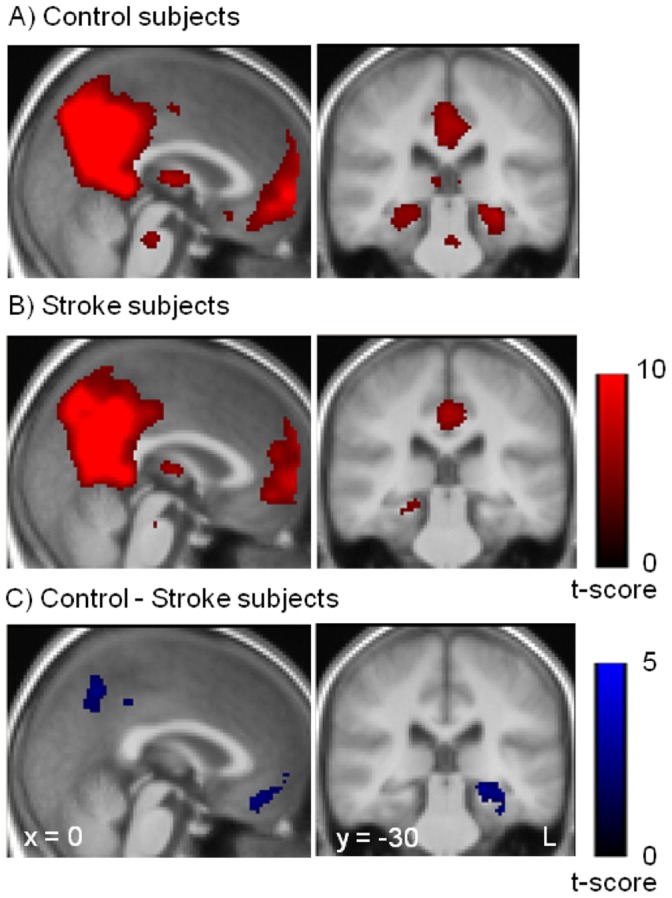
Resting state functional connectivity of the default mode. Spatial maps of the default mode network for control subjects (A) and for stroke subjects (B). (C) Resting state functional connectivity differences between control and stroke subjects. Stroke subjects showed decreased functional connectivity in the posterior cingulate gyrus, medial prefrontal cortex and left medial temporal lobe. The statistical maps are superimposed onto the spatially normalized and averaged group T1-images.

**Figure 3:**
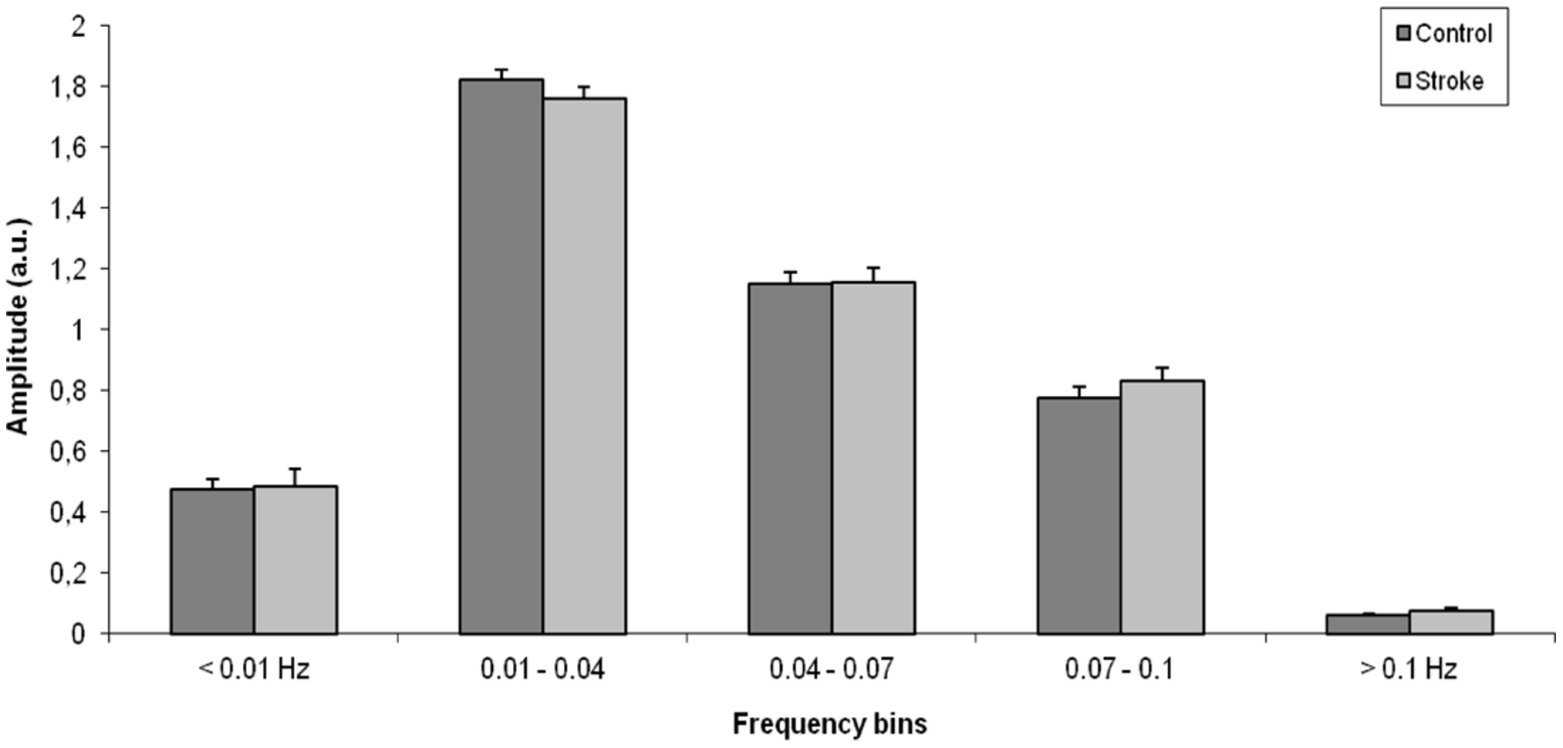
Average power spectrum (± SEM) of the time course of the independent component that belongs to the default mode network is shown for both control and stroke subjects. The highest power density was observed in the low frequency domain (0.01–0.04 Hz) for both groups. No significant differences in frequency characteristics were observed between the control and stroke subjects.

The group analyses of the independent components revealed seven distinct RSN’s (Fig. 1). These RSN’s resembles the RSN’s previously found in other studies [13], [30]. The component depicted by Fig. 2 had the highest spatial correlation with the DMN spatial template, which was generated by the WFU pick-atlas. The mean z-scores of the default mode network differed significantly between control subjects and patients (p<0.01, adjusted for age and gender). For the group comparison of the DMN (Fig. 2c), stroke subjects showed decreased functional connectivity in the medial prefrontal cortex, posterior cingulate cortex and left MTL. Decreased functional connectivity was also seen in the right medial temporal lobe with the same height threshold but with a lower extent threshold (figure not shown). An increased functional connectivity in stroke subjects is observed in the left lingual gyrus. Furthermore, a positive correlation was observed between the functional connectivity of the left MTL within the DMN and the performance of the delayed recall task of CVLT in control subjects (β = 0.700, p = 0.018), adjusted for age, gender and education. No correlation was however found in stroke subjects. Furthermore, no significant differences in frequency characteristics were observed between the control and stroke subjects (Fig. 3). As expected, we did not find any group differences for the control component reflecting the cerebellum.

### ROI-based Analyses

**Figure 4:**
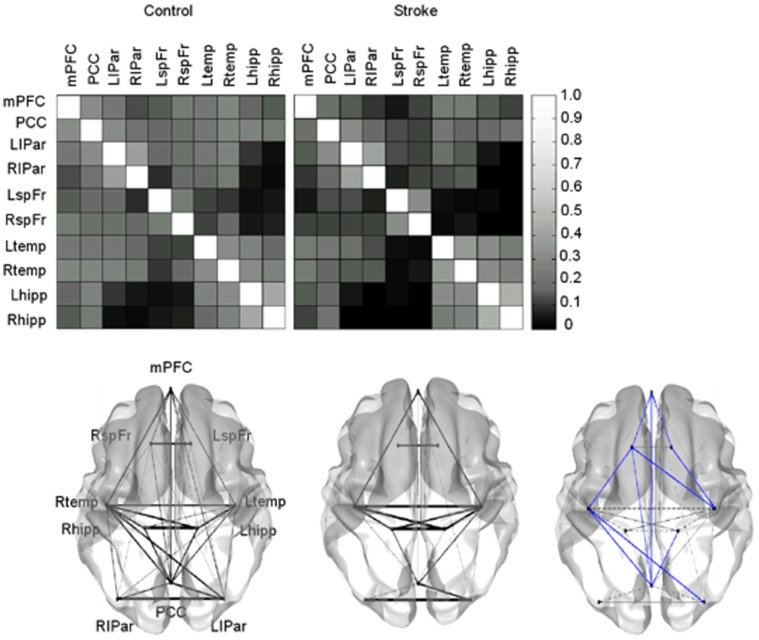
Region of interest (ROI) based analysis for the control and stroke subjects. The top panel displays the correlation coefficients matrices for every pair-wise region. The bottom panel displays the graph visualization of the correlation coefficient matrices thresholded at *r*>0.15. The bottom right represents the significant differences between control and stroke subjects; blue lines depicting stronger correlations in control with respect to stroke subjects at the threshold P<0.05, overlaid on the graph visualization of the stroke patients. In the opposite contrast, no stronger functional correlations were found in stroke subjects. mPFC = medial prefrontal cortex, PCC = posterior cingulate cortex, LIPar = left inferior parietal cortex, RIPar = right inferior parietal cortex, LspFr = left superior frontal cortex, RspFr = right superior frontal cortex, Ltemp = left middle temporal cortex, Rtemp = right middle temporal gyrus, LMTL = left medial temporal lobe, RMTL = right medial temporal lobe.

For every pair-wise region, the correlation coefficients were calculated after removing the non-neural BOLD fluctuations. This resulted in correlation matrices and corresponding graphs for visualization of the matrices for both control and stroke subjects (Fig. 4). The regions of the DMN in control subjects were strongly connected with each other, whereas a reduction of functional connectivity was observed in stroke subjects. Stroke patients showed a significantly reduced functional connectivity in several pair-wise regions, among others the posterior cingulate cortex, medial prefrontal cortex and the left MTL (p<0.05 uncorrected). Using FDR-correction for multiple comparisons, significant reduction in functional connectivity was found between right middle temporal cortex and right superior frontal cortex, and left middle temporal cortex and right superior frontal cortex in stroke patients when compared to control subjects. In the opposite contrast, there were no stronger correlations in stroke subjects compared to the control subjects. Correlation analyses using Pearson’s partial correlation revealed a non-significant positive trend between right temporal lobe and right superior frontal lobe (r = 0.43) and between left temporal lobe and right superior frontal lobe (r = 0.46) and a negative trend between posterior cingulum and right superior frontal lobe (r = −0.51) with the CVLT-delay recall score, adjusted for age, gender and education.

### Brain Morphology

There were no significant differences of the total intracranial, gray matter, white matter and CSF volume between control and stroke subjects (Table 3). VBM analyses revealed no statistically regional gray matter volume differences between the control and stroke subjects. Furthermore, the manual obtained hippocampal volumes did not differ significantly between the two groups.

**Table 3:** Volumetric data for the two study groups.

	Stroke (n = 20)	Control (n = 21)
Total intracranial volume, mL	1405 (±128)	1336 (±121)
Gray matter*	48 (±2.85)	49 (±2.85)
White matter*	30 (±2.51)	30 (±2.56)
Cerebrospinal fluid*	22 (±2.98)	21 (±3.87)
Left hippocampus*	0.24 (±0.03)	0.25 (±0.03)
Right hippocampus*	0.26 (±0.03)	0.26 (±0.03)

No significant differences in brain volumetry were found between stroke and control subjects. Values represent means (± SD).

* Values are percentage to the total intracranial volume (± SD).

## Discussion

We found a decreased functional connectivity in posterior cingulate, medial prefrontal and left MTL within the DMN in ‘first-ever’ stroke patients compared to the control subjects. In addition, reduced inter-regional functional connectivity between these regions was observed when comparing stroke patients with healthy control subjects.

Several methodological issues in this study need to be considered. A potential limitation of functional connectivity analyses is the contribution of physiological noise, such as cardiac and respiratory pulsations, on the functional connectivity [31]–[33]. Since we did not record the heartbeat and respiration pulsations during the scanning, we were not able to remove these fluctuations from our resting state data. However, we do not consider it plausible for these fluctuations to cause the differences we have found in the DMN in stroke patients. First, both the controls and patients were naïve to the MRI scanning and environment in the experimental setting. Second, we performed ICA that can separate the physiological noises from the neural network [13], [34]. Still, the residual effects of this noise may be present in the default mode component. Third, we confirmed the ICA results indirectly by applying ROI-based analyses in which we removed as much as possible the non-neural noises [33]. Second limitation is the use of ICA. We observed several significantly different regions within the DMN in the contrast control versus stroke subjects. This does not necessarily mean - though unlikely - that the connectivity is less in stroke patients, as independent component may not capture all variance of the data. To overcome this problem, we performed ROI-based analysis on the full time course. This analysis revealed decreased inter-regional functional connectivity in stroke patients, which supports indirectly the results of ICA. Third, the BOLD signal can be reduced in patients with stroke [35]. However, it seems unlikely to us that this could explain the results. No significant group-differences were found for the control component and the frequency distribution of the time course was not different between the groups. Furthermore, the hemodynamic response is normalized within few days after stroke [36]. Fourth, the presence of white matter lesions could affect the DMN activity, even though patients with low extent of white matter lesions were included in this study to reduce this possibility. Fifth, due to the cross-sectional nature of the study design, no causal relationship could be inferred. Also, the observed differences between the control subjects and stroke patients are small in regard with the multiple comparisons that have been carried out. The a prior hypothesis that the stroke patients would have reduced DMN activity, was tested using relatively liberal cut-off p-values. These values were selected in order to minimize the type II error and are commonly used in patient studies [6]. Also, a positive correlation has been found between functional connectivity in left MTL with CVLT delayed recall in control subjects and not in stroke patients. This could be explained by the small sample size of stroke population with various size and location of the infarction leading to differential influences on the default mode network. Another issue is that we selected ‘first-ever’ stroke patients irrespective of the size and location of the infarction. On the one hand, this study design limits the ability to the investigate the relationship between infarction location and whole-brain resting state connectivity, on the other hand this approach makes our results highly generalizable to the general stroke population. Strong elements of our study are that the alterations of the DMN in stroke patients were not caused by morphological changes in the cortical regions or by alterations of the frequency characteristics of the time course of the default mode component. There were also no differences in environmental factors such as anxiety and depression, concurrent general cognitive decline, or by pre-existing cognitive impairment as assessed by IQCODE, between the groups.

In terms of the interpretation of our findings, the altered functional connectivity is commonly considered to reflect the occurrence of the cognitive disturbances in various brain disorders [37]. DMN consists of regions that frequently exhibit a decrease in BOLD activity during goal-directed tasks [8] and show greater deactivation with increasing task difficulty [38]. During episodic memory retrieval, these regions are activated rather than deactivated [10], [39]. In the resting state condition, several studies showed reduction of DMN activity in patients with neurodegenerative diseases, who have reduced episodic memory function, when compared to the healthy control subjects [6], [11], [40]. Furthermore, MTL is a part of the DMN that has a key role in episodic memory processing [41]. This has been shown in healthy subjects and in patients with surgical removal of the MTL who are incapable of forming new episodic memories [42]. Another part of DMN is the posterior cingulate cortex that is related to the memory function and is believed to be a key node in the DMN [5]. These findings suggest that DMN is involved in episodic memory processing. In this study, stroke patients, who had reduced episodic memory function compared to healthy subjects, also have a reduction of the MTL and posterior cingulate cortex activity. A left-right asymmetry of decreased connectivity of the MTL was observed, with more decreased connectivity in the left MTL in stroke patients. This is in line with studies reporting the involvement of left MTL in verbal memory processing [43], [44]. Moreover, the degree of coactivation of left MTL correlated significantly with CVLT delayed recall score (a quantitative measure of the episodic memory) in control subjects. Although a non-significant positive trend was observed in stroke patients, the current results cannot fully support the notion that the disruption of DMN activity in stroke patients is related to episodic memory performance. One could argue that ‘normal’ episodic memory performers would show similar DMN activity when compared to control subjects. In the present study, however, we were unable to reliably investigate the differences between ‘normal’ with ‘impaired’ episodic memory performers, and with control subjects, due to the small sample size. Our findings, though, indirectly suggest that reduced DMN activity could be the underlying substrate of our finding of a reduced MTL functionality during the working-memory task in these stroke patients [4].

Theoretical explanations for widespread effects of local structural damage have been postulated for long time. This is referred to as ‘disconnection’ syndrome [45]. Recently, support for this theoretical idea comes from both computational lesion modeling studies and empirical studies in patients. Lesion modeling studies in human brain have shown that the impact of the lesions can have a profound effect on the spontaneous, anatomical remote ongoing BOLD activity that can result in widespread dynamical effects [46]. Results from patient studies also confirmed that the structural damage could lead to alterations of the functional connectivity pattern exerting its effect to other cortical regions [47], [48]. Also, several studies have shown that the resting state activity is correlated with post-stroke symptoms, such as motor problems [49], the degree of post-stroke depression [36] and behavioral performances [50]. In the latter study, authors found that the behavioral performances were correlated with the strength of the functional connectivity within the damaged hemisphere and more importantly, with the strength of the interhemispheric functional connectivity [50]. This supports the notion that lesions can affect the remote non-lesioned brain areas. As DMN is widely connected with others major hubs (regions with high number of connections) in the cortex [51], virtually every local structural damage in the brain can lead to the disruption of the DMN and the accompanying clinical symptoms. In a systematic review, this is further corroborated by the frequent finding of post-stroke memory dysfunction, without an association between the location of the stroke lesion and the occurrence of memory dysfunction [52]. Here, we observed that the DMN is different in stroke patients supporting the concept of disconnection syndrome. Stroke lesions might thus be responsible for the alterations of the DMN and, as such, could account for the underlying pathophysiological explanation for impaired cognitive performance, which cannot solely be explained by the location of the stroke lesion.

### Conclusion

This study complements the emerging evidence that infarctions not only result in focal neurological symptoms dependent on their location, but can also induce widespread effects in remote regions. Altered DMN connectivity in stroke patients might underlie reduced episodic memory after stroke. To further support these findings, future studies should incorporate stroke patients without impaired episodic memory function as extra validation. Future studies should also evaluate other resting state networks, which could be disrupted due to the effects of stroke lesions and should incorporate follow-up of patients in order to determine long-term consequences of such lesions on the functional and structural connectivity network and cognitive function, which can give us valuable information about the neural plasticity and functional recovery. This is important for the practitioners to identify stroke patients at risk for post-stroke dementia and to determine adequate medical treatment and/or cognitive or physical rehabilitation [53] that might be beneficial for the patients.
